# Weighted correlation gene network analysis reveals a new stemness index-related survival model for prognostic prediction in hepatocellular carcinoma

**DOI:** 10.18632/aging.103454

**Published:** 2020-07-09

**Authors:** Qiujing Zhang, Jia Wang, Menghan Liu, Qingqing Zhu, Qiang Li, Chao Xie, Congcong Han, Yali Wang, Min Gao, Jie Liu

**Affiliations:** 1 Department of Oncology, Shandong Cancer Hospital and Institute, Shandong First Medical University and Shandong Academy of Medical Sciences, Jinan 250117, Shandong, China; 2 Department of Oncology, Zibo Maternal and Child Health Hospital, Zibo 255000, Shandong, China; 3 Basic Medicine College, Shandong First Medical University, Taian 271016, Shandong, China; 4 Department of Oncology, Mengyin County Hospital, Linyi 276299, Shandong, China; 5 Department of Radiotherapy, Shandong Cancer Hospital and Institute, Shandong First Medical University and Shandong Academy of Medical Sciences, Jinan 250117, Shandong, China

**Keywords:** hepatocellular carcinoma, mRNA expression-based stemness index, survival model, weighted correlation network analysis, The Cancer Genome Atlas

## Abstract

In this study, we constructed a new survival model using mRNA expression-based stemness index (mRNAsi) for prognostic prediction in hepatocellular carcinoma (HCC). Weighted correlation network analysis (WGCNA) of HCC transcriptome data (374 HCC and 50 normal liver tissue samples) from the TCGA database revealed 7498 differentially expressed genes (DEGs) that clustered into seven gene modules. LASSO regression analysis of the top two gene modules identified *ANGPT2*, *EMCN*, *GLDN*, *USHBP1* and *ZNF532* as the top five mRNAsi-related genes. We constructed our survival model with these five genes and tested its performance using 243 HCC and 202 normal liver samples from the ICGC database. Kaplan-Meier survival curve and receive operating characteristic curve analyses showed that the survival model accurately predicted the prognosis and survival of high- and low-risk HCC patients with high sensitivity and specificity. The expression of these five genes was significantly higher in the HCC tissues from the TCGA, ICGC, and GEO datasets (GSE25097 and GSE14520) than in normal liver tissues. These findings demonstrate that a new survival model derived from five strongly correlating mRNAsi-related genes provides highly accurate prognoses for HCC patients.

## INTRODUCTION

The incidence of new cases of liver cancer increased by 2% to 3% annually between 2007 and 2016, according to the cancer statistics reported in 2020 [1]. The mortality rate of liver cancer ranks second among all the cancers worldwide, and the five-year survival rate is only 18% [2]. Hepatocellular carcinoma (HCC) is the most common primary liver cancer that accounts for nearly 90% of all liver cancer patients [3]. The standard therapy for HCC is surgical resection [4]. However, most patients are not amenable for surgical resection therapy because of disease progression and extrahepatic metastasis [5]. Furthermore, the five-year recurrence rate after surgical resection is 70% for HCC, with tumor recurrence reported in nearly two-thirds of the patients within two years after surgery [6]. Moreover, the sensitivity or specificity of current diagnostic imaging and tumor biomarkers such as α-fetoprotein (AFP), Protein induced by vitamin K absence-II (PIVKA-II), and Des-gamma carboxyprothrombin (DCP) is extremely low and cannot detect early stages of HCC accurately [7]. Therefore, there is an urgent need to identify reliable prognostic models for early diagnosis and accurate prognosis of HCC.

Tumorigenesis involves malignant cells acquiring stem cell-like characteristics, including self-renewal and differentiation [8]. Malta et al*.* used a machine learning algorithm to quantify the stemness index of tumors based on their dedifferentiation characteristics; they also demonstrated that the stemness index correlates with the survival times of HCC patients [9]. The application of New Generation Sequencing (NGS) technology and open access to major databases has resulted in identification of several potential prognostic and early diagnostic biomarkers in HCC, including *Protocadherin 19* (*PCDH19*) gene hypermethylation [10], *Glypican-3* or *GPC3* [11] and *Cytochrome P450 Family 3 Subfamily A Member 4* or *CYP3A4* [12]. Moreover, the overexpression of *YTH N6-Methyladenosine RNA Binding Protein 1* or *YTHDF1* [13] and *DDB1 and CUL4 associated factor 13* or *DCAF13* [14] is associated with poor prognosis of HCC. However, the biological role of key genes that determine the stemness index in HCC has not been reported so far. Furthermore, recent studies have identified several potential prognostic biomarker genes based on differential expression in HCC [15, 16], but their mechanistic role remains to be investigated in greater detail. Weighted correlation network analysis (WGCNA) is a method that identifies gene modules (GMs) containing highly correlating genes with potentially similar biological functions [17]. It has been widely used in the identification of disease characteristics, cancer-related biomarkers and therapeutic target genes of several cancers, such as non-small cell lung cancer [18], rectum adenocarcinoma [19], uveal melanoma [20], bladder cancer [21], and clear cell renal cell carcinoma [22, 23]. Therefore, in this study, we used WGCNA to classify DEGs with closely related stemness index into GMs in HCC. Then, we identified five key genes linked to mRNA expression-based stemness index (mRNAsi) with similar biological characteristics using the least absolute shrinkage and selection operator (LASSO) regression analysis. Furthermore, we developed a survival model using these five genes and evaluated prognostic prediction accuracy of these mRNAsi-related genes in HCC patients. To our knowledge, this is the first time that WGCNA has been used to screen key mRNAsi-related genes and build a survival model to predict prognosis of HCC.

## RESULTS

### Identifying mRNAsi-related DEGs in HCC

**Figure 1:**
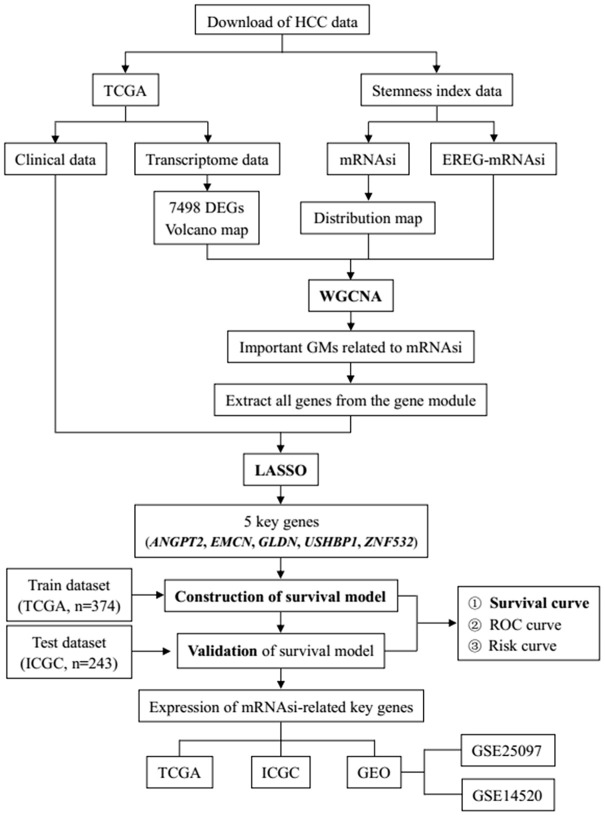
**The flowchart of HCC data preparation, processing, analysis and validation.**

**Figure 2:**
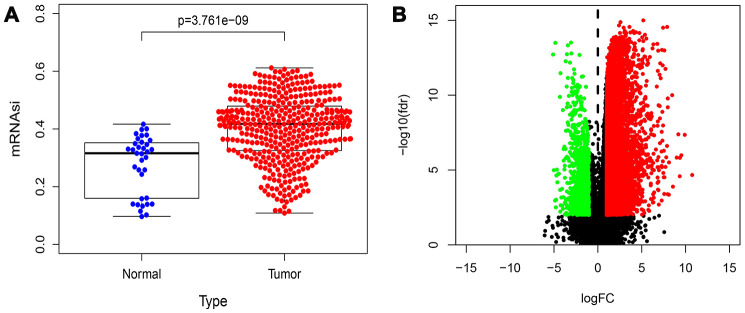
**Distribution map of mRNAsi and DEGs in HCC.** (**A**) Distribution map shows the mRNAsi of genes in HCC and control samples from the study published by Malta et al. The X axis is sample type (Normal or Tumor) and the Y axis is mRNAsi. (**B**) The volcano plot shows the expression profiles of 7498 DEGs in HCC samples compared to normal liver samples from the TGCA database. The low expressing genes (n=394) are shown in green and the high expressing genes (n=7104) are shown in red. The threshold criteria are FDR/fdr=0.01 and log_2_FC=1.

Figure 1 shows the flowchart of data analysis in this study. We analyzed the mRNAsi status of genes expressed in HCC samples as previously reported by Malta et al [9] and found that the mRNAsi were significantly higher in the HCC tumor samples compared to the normal liver tissue samples (p=3.761e−09; Figure 2A). Then, we used the edgeR software to analyze the transcriptome of 374 HCC and 50 normal liver tissue samples from The Cancer Genome Atlas (TCGA) database and identified 7498 DEGs in HCC tumor tissues relative to normal liver tissues (Supplementary Table 1). The volcano plot in Figure 2B depicts the genes that are expressed significantly higher (red) or lower (green) in the HCC tumor tissues relative to normal liver tissues, including 7104 genes with high expression and 394 genes with low expression.

### Identification of gene modules among DEGs in HCC using WGCNA

**Figure 3:**
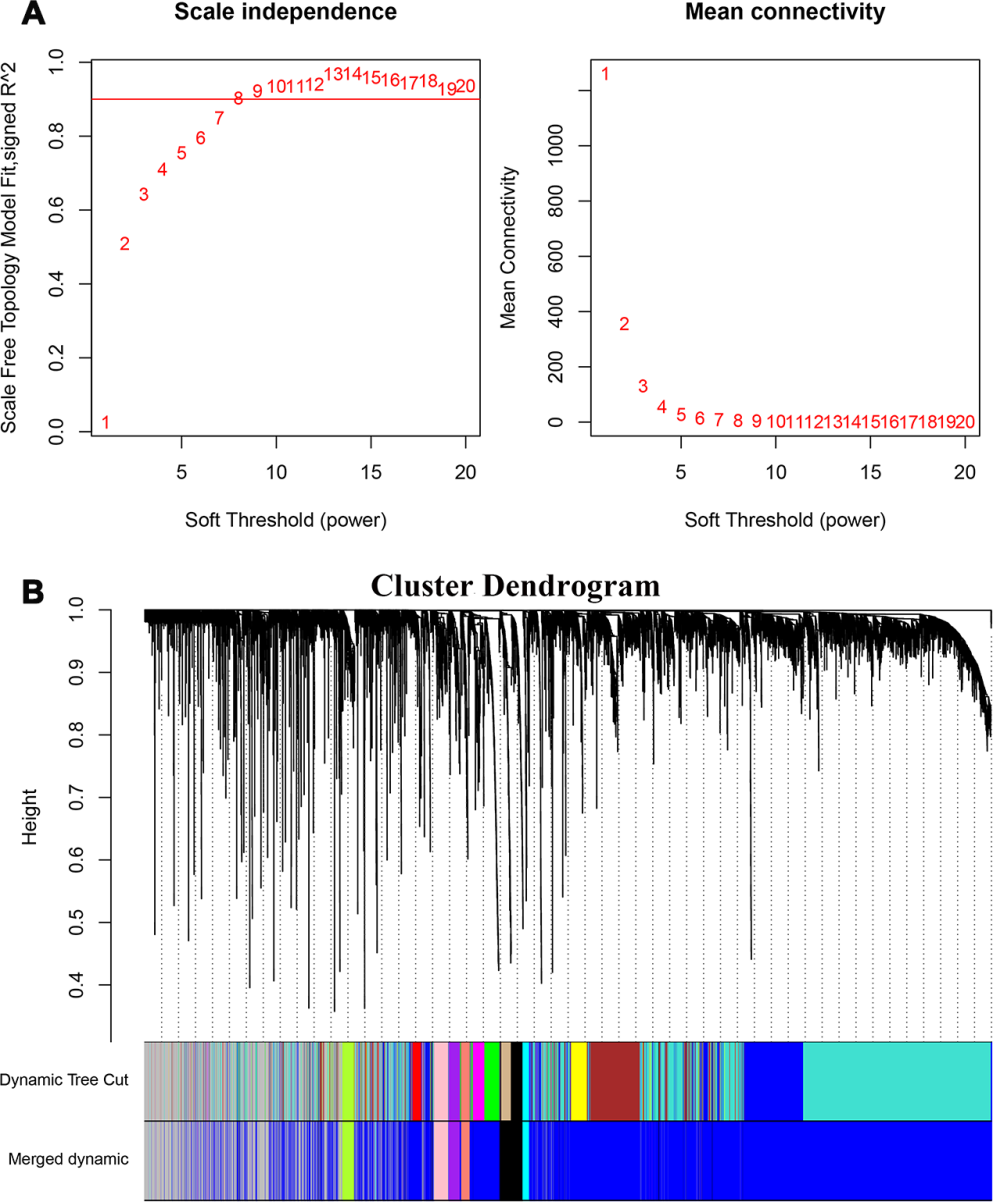
**Weighted gene co-expression network analysis of HCC transcriptome.** (**A**) The graph shows the scale-free fit index for various soft threshold powers to identify the optimal soft threshold power (β). In the graph on the left, the horizontal axis represents the soft threshold power or β values and the vertical axis represents the scale-free network index (R^2^). The scale-free characteristics of the gene network are stronger when the R^2^ value is higher. In the right graph, the horizontal axis represents the soft threshold power or β values, the vertical axis represents the means of all the gene adjacency functions in the corresponding gene module. (**B**) Identification of co-expressed gene modules in HCC. The different branches of the cluster dendrogram correspond to different gene modules that are represented by different colors. Each piece of the leaves corresponds to a single gene in the module.

**Figure 4:**
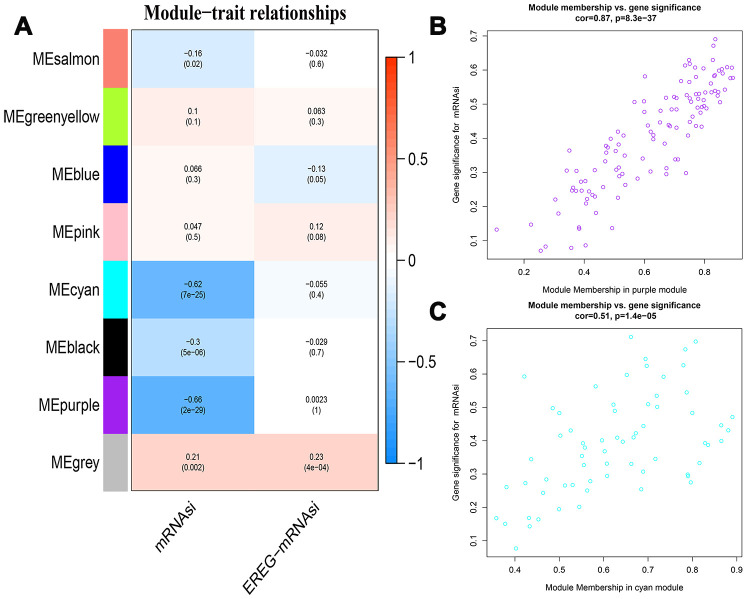
**Identification of modules associated with stemness index in HCC.** (**A**) The table shows the module-trait relationships of all gene modules, which are represented by different colors. Each cell in the table shows the correlation co-efficient and the p-value between the gene module in rows and the mRNAsi or EREG-mRNAsiin the columns. The degree of correlation is indicated by the color depth; red represents a positive correlation and blue represents a negative correlation. (**B**, **C**) The scatter plots of genes in the top 2 gene modules, purple (**B**, n=116) and cyan (**C**, n=44). The X axis is module membership in modules and the Y axis is gene significance for mRNAsi.

The 7498 DEGs combined with stemness index data were then analyzed using WGCNA with a soft threshold power (β) value of 8 (Figure 3A). Then, we constructed a cluster dendrogram that grouped co-expressing genes into seven gene modules (GMs) that are shown in different color codes, as analyzed using the hybrid dynamic cutting tree algorithm in Figure 3B. Then, we analyzed the module significance (MS) value by evaluating the correlation between each module and the mRNAsi or epigenetically regulated mRNAsi (EREG-mRNAsi). The modules showing a higher correlation value were ranked higher, thereby indicating the higher significance of the module. As shown in Figure 4A, the degree of correlation is indicated by the color depth and the color codes indicate positive (red) or negative (blue) correlation of the modules to the mRNAsi and the EREG-mRNAsi. Among the seven GMs, the purple module (n=116 DEGs) showed the highest correlation of 0.7 with the mRNAsi, followed by the cyan module (n=44 DEGs) with a correlation co-efficient 0.62. Hence, we chose the purple and cyan modules for further analyses. We constructed a scatter diagram to display the genes in these two modules based on the gene significance (GS) and the module membership (MM) of each gene (Figure 4B, 4C), The X axis in the scatter diagram is MM in modules and the Y axis is GS for mRNAsi. The details of the genes in the scatter diagram are shown in Supplementary Table 2A, 2B.

### The construction of survival model

We performed univariate Cox regression and LASSO regression analyses of the mRNAsi-related genes from the purple and cyan GMs and identified five key genes, *Angiopoietin 2* (*ANGPT2*), *Endomucin* (*EMCN*), *Gliomedin* (*GLDN*), *USH1 Protein Network Component Harmonin Binding Protein 1* (*USHBP1*) and *Zinc Finger Protein 532* (*ZNF532*), which were then used to construct the survival model (Table 1). The risk score for HCC patients based on this survival model was calculated according to the following formula: (0.154×*ANGPT2*) + (−0.138×*EMCN*) + (0.043×*GLDN*) + (−0.265×*USHBP1*) + (0.121×*ZNF532*). Each gene stands for the gene expression in the gene transcriptome data, and the number represents the model co-efficient of each gene.

**Table 1:** The LASSO regression analysis results.

**Gene**	**Co-efficient**
ANGPT2	0.154
EMCN	-0.138
GLDN	0.043
USHBP1	-0.265
ZNF532	0.121

LASSO: the least absolute shrinkage and selection operator; ANGPT2: Angiopoietin 2; EMCN: Endomucin; GLDN: Gliomedin; USHBP1: USH1 Protein Network Component Harmonin Binding Protein 1; ZNF532: Zinc Finger Protein 532.

### Verification of survival model

**Figure 5:**
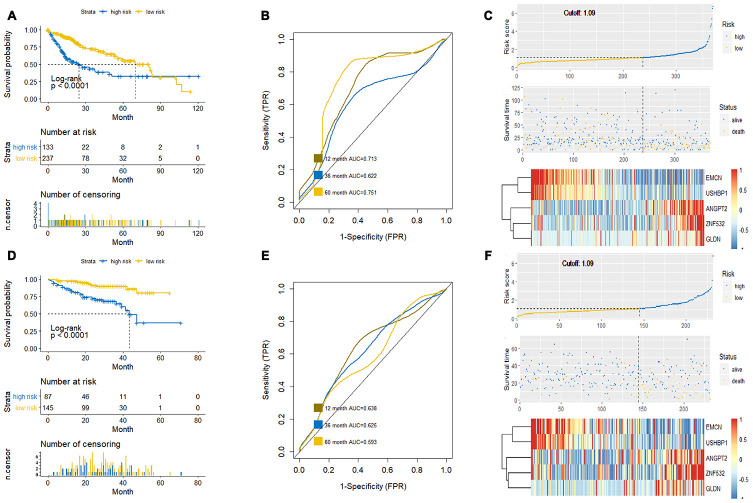
**Verification of the prognostic prediction accuracy of the new survival model.** (**A**, **C**) The Kaplan-Meier survival curve (**A**), ROC curve (**B**) and Risk curve (**C**) analyses of the high-risk and low-risk HCC patients of the training dataset from the TCGA database based on the new survival model is shown. (**D**–**F**) The Kaplan-Meier survival curve (**D**), ROC curve (**E**) and Risk curve (**F**) of the high-risk and low-risk HCC patients in the test dataset from the ICGC database based on the new survival model is shown. The horizontal axis of the Kaplan-Meier survival curve is survival time (month) and the vertical axis is patient survival, which is used to evaluate the prognostic prediction ability of the new model (P < 0.05 is considered to be statistically significant); the ROC curve evaluates the sensitivity and specificity of the model, in which the Abscissa is the specificity of the model and the ordinate is the sensitivity; moreover, the risk curve shows that the risk of death increases with the increase of the risk score of the new survival model.

Next, we used the HCC tumor sample data from the TCGA database (training dataset) to verify if the prognostic prediction of this new survival model was accurate, specific and sensitive. We generated Kaplan-Meier survival curves, receiver operating characteristic (ROC) curve and the risk curve of high and low risk groups, which were classified based on the risk score formula of this new survival model (Figure 5A–5C). We observed that the difference in survival between high and low risk groups is statistically significant (p<0.0001; Figure 5A). Furthermore, ROC curve analysis showed that the survival model composed of *ANGPT2*, *EMCN*, *GLDN*, *USHBP1* and *ZNF532* showed good predictive value for survival when analyzed at 12 months (area under the curve (AUC)=0.713), 36 months (AUC=0.622), and at 60 months (AUC=0.751) for the HCC patients (Figure 5B). The risk curve indicated that the death toll of HCC patients increases with the increase of risk score (Figure 5C). Furthermore, we verified the new survival model in a test dataset of HCC patients from the International Cancer Genome Consortium (ICGC) database (Figure 5D-5F). The survival curve analysis showed statistically significant results in distinguishing high and low risk patient groups of the test dataset (Figure 5D). Moreover, ROC curve analyses showed good predictive value for survival with AUC values of 0.638, 0.625, and 0.593 at 12, 36 and 60 months, respectively (Figure 5E, 5F). The risk curve of the test dataset also showed the same trend with the training dataset from TCGA (Figure 5F). These results showed that the survival model constructed by mRNAsi-related genes, *ANGPT2*, *EMCN*, *GLDN*, *USHBP1* and *ZNF532* accurately predicted the survival of HCC patients.

### The expression of the five survival model genes in HCC patient samples

**Figure 6:**
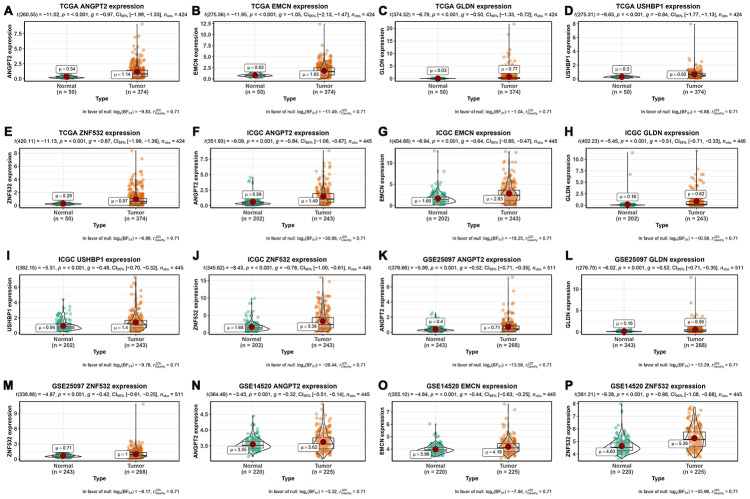
**Expression of mRNAsi-related key genes in HCC and normal liver tissues.** (**A**–**E**) The expression of *ANGPT2* (A), *EMCN* (B), *GLDN* (**C**), *USHBP1* (**D**) and *ZNF532* (**E**) genes in 374 HCC and 50 non-cancer tissues from the TCGA database. (**F**–**J**) The expression of *ANGPT2* (**F**), *EMCN* (**G**), *GLDN* (**H**), *USHBP1* (**I**) and *ZNF532* (**J**) genes in 243 HCC and 202 normal liver tissues from the ICGC database. (**K**–**M**) The expression of *ANGPT2* (**K**), *GLDN* (**L**) and *ZNF532* (**M**) genes in 268 HCC and 243 normal liver tissue samples from the GSE25097 dataset. (**N**–**P**) The expression of *ANGPT2* (**N**), *EMCN* (**O**) and *ZNF532* (**P**) genes in 225 HCC and 220 normal liver tissue samples from the GSE14520 dataset. The X axis is sample type (Normal or Tumor) and the Y axis is gene expression.

**Figure 7:**
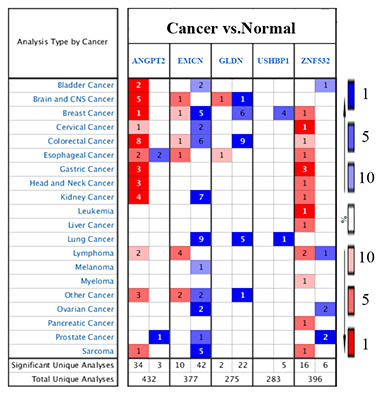
**The expression of five survival model genes in various cancers in the Oncomine database.** The expression of *ANGPT2,* *EMCN*, *GLDN*, *USHBP1*, and *ZNF532* genes in the tumor and control samples of different cancers (bladder cancer, breast cancer, cervical cancer, colorectal cancer, esophageal cancer, gastric cancer, head and neck cancer, kidney cancer, leukemia, liver cancer, lung cancer, lymphoma and other cancers) in the Oncomine database are shown.

Finally, we analyzed the expression of the five survival model genes using HCC patient data in the TCGA and ICGC databases, which were used as the training and test datasets, respectively. The results showed that the expression of all the five genes was significantly higher in the HCC tissues from both the databases compared to the adjacent normal liver tissues (p<0.001; Figure 6A–6J). Moreover, we analyzed the expression of these genes in the GSE25097 and GSE14520 datasets from the Gene Expression Omnibus (GEO) database. The GSE25097 dataset of 557 samples included 268 HCC, 243 adjacent non-tumor liver tissues, 40 cirrhotic and 6 healthy liver samples. The expression of *ANGPT2*, *GLDN*, *and ZNF532* was significantly higher in the 268 HCC tumor tissues of the GSE25097 dataset compared to the 243 non-tumor liver tissue samples (p < 0.001; Figure 6K–6M). However, the expression of *EMCN* and *USHBP1* genes was similar in both HCC and adjacent normal liver tissues samples (Supplementary Figure 1). The GSE14520 dataset (225 HCC tumor tissues and 220 liver non-tumor tissues) lacked the data for *GLDN* and *USHBP1* expression, but the expression of the other three genes *ANGPT2* (p=0.001), *EMCN* (p < 0.001), *ZNF532* (p < 0.001) were significantly higher in the HCC tissues compared to the adjacent normal liver tissue samples (Figure 6N–6P). As shown in Figure 7, we analyzed the patient sample data of other cancers (bladder cancer, breast cancer, cervical cancer, colorectal cancer, esophageal cancer, gastric cancer, head and neck cancer, kidney cancer, leukemia, liver cancer, lung cancer, lymphoma, etc.) in the Oncomine database and found that the expression of *ANGPT2*, *EMCN*, *GLDN*, *USHBP1*, *ZNF532* gene was significantly upregulated in most tumor tissue samples compared to the adjacent normal tissue samples.

## DISCUSSION

HCC is a highly malignant cancer with high morbidity and mortality rates [22]. Currently, there is an urgent need to identify new molecular biomarkers that can improve early diagnosis as well as accurate prognosis prediction that can guide appropriate treatment to improve survival rates. Although several prognostic and diagnostic biomarkers have been reported for HCC, their reliability and efficacy remain to be verified for clinical applications. Moreover, the previous prognostic models ignore the correlation between genes. A recent study by Malta et al demonstrated the correlation between mRNAsi-related genes and the survival and prognosis of cancer patients in all TCGA tumors [9]. However, mRNAsi-related molecular markers have not been reported for HCC. Therefore, we performed WGCNA analysis of the microarray data of HCC patients and identified gene modules (GMs) with mRNAsi-related genes. Besides, LASSO regression analysis of the genes in the top 2 GMs identified five key genes, which were then used to construct the new survival model of HCC. Our study suggests that these 5 genes are potential prognostic and therapeutic targets for HCC. However, future investigations are necessary to demonstrate the clinical significance of these genes.

WGCNA is an algorithm that clusters genes with similar patterns of expression into GMs [17]. This allows establishing the correlation between GMs and the characteristics of patient samples in different stages of progression. Thus, WGCNA has been used extensively to study the prognostic potential of several genes that correlate with patient prognosis and survival [24].

In this study, we first identified 7498 HCC-related DEGs and used WGCNA to classify them into seven gene modules based on their correlation with the mRNAsi. Furthermore, genes in the purple module and cyan module showed the highest correlation with the mRNAsi. We then identified 5 key mRNAsi-related genes from these two models using LASSO regression analysis and then constructed a survival model with these five genes to predict the prognosis and survival of HCC patients. Then, we successfully verified that the survival model accurately predicts the prognosis of HCC patients by using patient’s data from the TCGA and ICGC databases as the training and test groups, respectively. We also found that the expression of these 5 genes, namely, *ANGPT2*, *EMCN*, *GLDN*, *USHBP1* and *ZNF532*, was significantly upregulated in HCC tumor tissues compared to the adjacent normal liver tissues in the TCGA and ICGC datasets. We also verified the survival model using GSE25097 and GSE14520 datasets. The expression of *EMCN* and *USHBP1* was not statistically significant in the HCC patients compared to the controls from the GSE25097 dataset, but the expression of *ANGPT2*, *GLDN* and *ZNF532* was significantly higher than the controls. The reason for this discrepancy is not known and needs to be evaluated in future studies. On the other hand, the GSE14520 dataset lacked expression data for the *GLDN* and *USHBP1* genes. Nevertheless, the expression of *ANGPT2*, *EMCN* and *ZNF532* genes was significantly higher in the HCC tumor samples compared to the normal liver tissue samples. Furthermore, analysis of the expression profiles of these five genes in the Oncomine database demonstrated differential expression in several cancers. However, these 5 genes were not differentially expressed in the liver cancer samples of the Oncomine dataset. One plausible reason for this anomaly is that the liver cancer samples in the large Oncomine database may belong to different pathological types of liver cancer and therefore represents a heterogeneous dataset. Another plausible reason is that the threshold setting we used may not be appropriate for screening samples in the Oncomine database. Overall, our data suggests that the survival model constructed using the *ANGPT2*, *EMCN*, *GLDN*, *USHBP1* and *ZNF532* genes shows good predictive value and demonstrates potential for clinical use to evaluate the prognosis of patients with HCC.

An integrated analysis of genomic and expression profiling found that the high expression of *nucleophosmin* (*NPM1*) in HCC was associated with the prognosis of patients [25]. It is plausible that gene copy number variations may also influence the prognosis and survival of HCC patients. However, gene copy number variations of these 5 survival model genes need to be evaluated in the HCC patients.

As far as we know, except for *ANGPT2,* the remaining four genes have not been previously identified as biomarkers for HCC patients. *ANGPT2* encodes for the angiopoietin-2 protein, which competitively inhibits angiopoietin-1 by specifically binding to the angiopoietin receptor, and thereby modulates the growth and progression of several cancers [26–28]. A prospective study shows that angiogenesis-related genes, including *ANGPT2*, are independent factors that correlate with the tumor progression and prognosis of liver cancer patients [29]. Chen et al showed that serum *ANGPT2* levels represent a potential serum prognostic biomarker in liver cancer patients [30]. *ANGPT2* is an essential factor for the formation of vessels that encapsulate tumor clusters (VETC), which is a unique vascular pattern that is associated with HCC progression [31].

*EMCN* encodes a type I O-glycosylated sialic acid-rich glycoprotein called endomucin I, which is specifically expressed on the endothelial cells of veins and capillaries [32]. Endomucin I is a novel therapeutic target for angiogenesis-related diseases because it inhibits vascular endothelial growth factor (VEGF)-induced migration, growth and morphogenesis of endothelial cells by modulating vascular endothelial growth factor receptor 2 (VEGFR2) endocytosis and activity [33, 34]. Moreover, a study by Holmfeldt et al*.* identified *EMCN* as one of the 17 genes that regulates repopulation of murine hematopoietic stem cells [35].

*GLDN* is located on chromosome 15 and its protein product promotes the adhesion of heterogeneous cells by selectively binding to the extracellular protein complexes [36]. *GLDN* is a potential prognostic biomarker that predicts the overall survival (OS) of patients with colorectal cancer [37] and melanoma patients that may benefit from immunotherapy [38].

*USHBP1* gene, also known as *MCC2* gene, is expressed in the heart, liver, small intestine, lung and other tissues [39]. A Genome-Wide Association Study (GWAS) study by Hass et al showed that *USHBP1* was involved in schizophrenia by regulating synaptic tissue development [40]. *ZNF532* encodes a protein that prominently interacts with the BRD4-NUT interacting fusion oncoprotein in the chromatin of NUT midline carcinoma cells and drives oncogenesis by propagating the oncogenic chromatin complex [41, 42].

WGCNA has recently been used to identify new gene targets that regulate gene progression for HCC prognosis and therapy [43, 44, 24]. Although mRNAsi has been shown to be related to prognosis and survival of HCC patients [9], the mRNAsi-related prognostic markers have not been studied. We used WGCNA algorithm to screen HCC-related mRNAsi genes for the first time and successfully constructed and verified a new survival model that can predict the prognosis of HCC patients. This prognostic model needs to be further confirmed using prospective multicenter randomized controlled trials. Moreover, the mechanism details of the five genes that have been used to develop this survival model needs to be further explored in HCC.

In conclusion, our study used WGCNA and LASSO regression analyses to identify five mRNAsi-related genes, namely, *ANGPT2*, *EMCN*, *GLDN*, *USHBP1* and *ZNF532*. We then constructed a survival model with these five genes and successfully verified their accuracy, sensitivity and specificity to predict the prognosis of HCC patients in TGCA, ICGC and GEO databases. We postulate that these five survival model genes are potential therapeutic targets of HCC.

## MATERIALS AND METHODS

### HCC data download and processing

We downloaded the transcriptome and clinical data of 374 HCC and 50 paracancerous patient samples from the TCGA [45] database (https://portal.gdc.cancer.gov) using "TCGA-LIHC" (TCGA-Liver hepatocellular carcinoma) as the project id, "liver and intrahepatic bile ducts" as the primary site, and "HTSeq-FPKM" as the workflow type on December 18, 2019. The sample identifiers of the TCGA data are shown in Supplementary Table 3. The stemness index data for HCC, including their mRNAsi and EREG-mRNAsi was downloaded from the study published by Malta et al [9] and is listed in Supplementary Table 4. After downloading the mRNAsi data, we analyzed the distribution of the mRNAsi in the normal and HCC samples. Then, we used the edgeR software package version: 3.26.5 [46] to clean and filter the downloaded transcriptome data of HCC. Finally, the DEGs between the normal and HCC samples was obtained using the following threshold parameters: false discovery rate (FDR) = 0.01 and log_2_ fold change in gene expression (FC) = 1.

### Gene module construction using WGCNA

WGCNA [17] was used to perform co-expression scale-free network analysis and identify gene modules containing strongly correlating genes. We imported the DEGs into the WGCNA software R package version: 1.68 [47] and determined that the soft power value was 0.8 based on the scale-free topology fit model index (R^2^), which was achieved along with a mean connectivity value below 100. Then, the difference between a pair of genes was calculated using the topological overlap method to construct the cluster dendrogram. We then re-analyzed the module eigengenes (MEs) according to the standard of the hybrid dynamic cutting tree and merged two or more modules that were close to each other into a new module.

We used the gene significance (GS) index to determine the strength of the correlation between every single gene and the mRNAsi or EREG-mRNAsi. We also used the module membership (MM) value to measure the importance of genes in the corresponding modules. The method to obtain GS is use the modeEigengenes function in WGCNA software package to calculate the characteristic genes of the module firstly, then take the correlation value between the expression of DEGs and the module eigengenes (MEs) as the GS. In addition, MM is calculated by taking the correlation between the expression of DEGs and the mRNAsi or EREG-mRNAsi of the corresponding samples downloaded so that GS and MM be accurately assigned to each gene in the module. The module significance (MS) of each module was determined by calculating the GS between sample traits (mRNAsi or EREG-mRNAsi) and gene expression. Subsequently, P < 0.05 was used as the statistically significant standard to screen important GMs. Finally, scatter diagram was constructed based on the correlation between GS and MM in the top 2 GMs to identify the key genes.

### Survival model construction

We performed univariate Cox hazard analysis [48] with P < 0.05 as a threshold parameter for all the genes in the top 2 GMs. Then, the lambda value with the minimum average error obtained from the cross-validation method was fitted into the LASSO regression analysis [49] to obtain key genes related to mRNAsi. These key genes were then used to construct the survival model of HCC. We determined the risk scores based on the expression of key genes in the 374 HCC tumor samples downloaded from TCGA database (training dataset), and grouped all the samples into high- and low-risk groups based on the scores. Then, we used the clinical information of these HCC patients in the high- and low-risk groups to generate the Kaplan-Meier survival curve and the ROC curves to determine the survival parameters as well as the AUC value, respectively, in order to determine the prognostic performance of the survival model.

### Verification of survival model

To independently verify the reliability of the survival model, we downloaded the transcriptome data and clinical information of 202 normal paracancerous samples and 243 HCC samples on November 27, 2019 from the LIRI-JP (https://dcc.icgc.org/releases/current/Projects/LIRI-JP) project in the ICGC database version: release_28 (https://icgc.org/). The sample identifiers of ICGC data are shown in Supplementary Table 5. The 243 HCC samples were selected as the test dataset and were analyzed similar to the training dataset as described above.

### Expression of the five survival model genes in different datasets

We used the cowplot (version: 1.0.0) and Ggstatsplot (version: 0.1.3) software packages to determine the expression of key mRNAsi-related genes that are included in the survival model in two randomly selected HCC patient datasets, GSE25097 and GSE14520 in the GEO (http://www.ncbi.nlm.nih.gov/geo/) database [50]. There were 268 and 225 HCC samples, 243 and 220 normal liver tissue samples in GSE25097 dataset and GSE14520 dataset, respectively. Furthermore, we retrieved the expression of these five mRNAsi-related genes in several cancer types from the Oncomine (http://www.oncomine.org) database [51] on December 24, 2019. We used "Cancer vs. Normal Analysis" as the analysis type and "p-value = 1E-4, FC = 2, gene rank = top 10%, and data type = all" as the threshold parameters.

## Supplementary Material

Supplementary Figure 1

Supplementary Table 1

Supplementary Table 2

Supplementary Table 3

Supplementary Table 4

Supplementary Table 5
