# Supplementary material for: Weighted correlation gene network analysis reveals a new stemness index-related survival model for prognostic prediction in hepatocellular carcinoma
Source: Aging (Albany NY). 2020 Jul 9;12(13):13502–17. doi: 10.18632/aging.103454 (PMC7377834; doi:10.18632/aging.103454)
Supplement: Supplementary Figure 1 [file aging-12-103454-s006..pdf]

SUPPLEMENTARY FIGURE

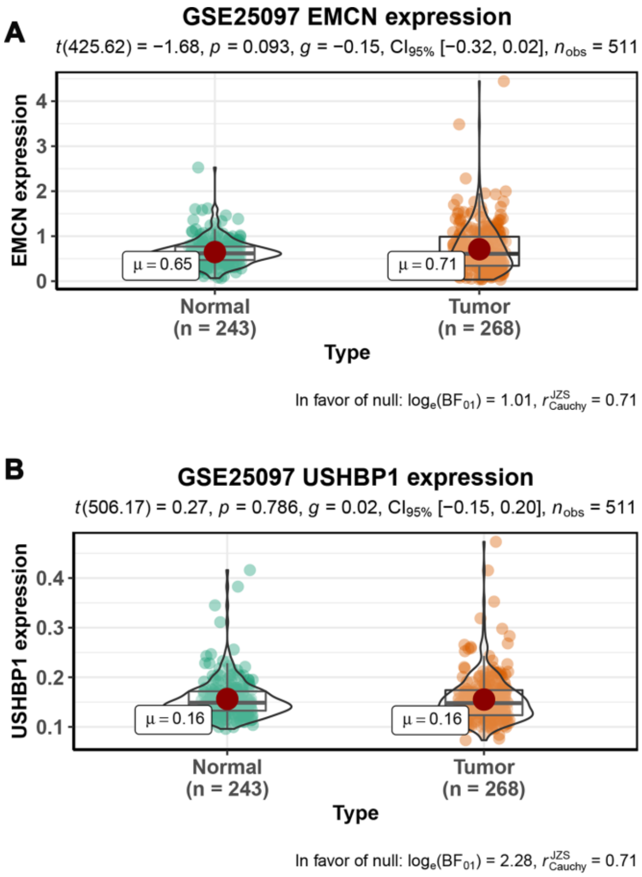

Supplementary Figure 1. The expression of *EMCN* and *USHBP1* in HCC and control samples in the GSE25097 dataset.
